# Validation of deep-learning-based triage and acuity score using a large national dataset

**DOI:** 10.1371/journal.pone.0205836

**Published:** 2018-10-15

**Authors:** Joon-myoung Kwon, Youngnam Lee, Yeha Lee, Seungwoo Lee, Hyunho Park, Jinsik Park

**Affiliations:** 1 Department of Emergency Medicine, Mediplex Sejong Hospital, Incheon, Korea; 2 VUNO, Seoul, Korea; 3 Department of Cardiology, Mediplex Sejong Hospital, Incheon, Korea; Duke-NUS Medical School, SINGAPORE

## Abstract

**Aim:**

Triage is important in identifying high-risk patients amongst many less urgent patients as emergency department (ED) overcrowding has become a national crisis recently. This study aims to validate that a Deep-learning-based Triage and Acuity Score (DTAS) identifies high-risk patients more accurately than existing triage and acuity scores using a large national dataset.

**Methods:**

We conducted a retrospective observational cohort study using data from the Korean National Emergency Department Information System (NEDIS), which collected data on visits in real time from 151 EDs. The NEDIS data was split into derivation data (January 2014-June 2016) and validation data (July-December 2016). We also used data from the Sejong General Hospital (SGH) for external validation (January-December 2017). We predicted in-hospital mortality, critical care, and hospitalization using initial information of ED patients (age, sex, chief complaint, time from symptom onset to ED visit, arrival mode, trauma, initial vital signs and mental status as predictor variables).

**Results:**

A total of 11,656,559 patients were included in this study. The primary outcome was in-hospital mortality. The Area Under the Receiver Operating Characteristic curve (AUROC) and Area Under the Precision and Recall Curve (AUPRC) of DTAS were 0.935 and 0.264. It significantly outperformed Korean triage and acuity score (AUROC:0.785, AUPRC:0.192), modified early warning score (AUROC:0.810, AUPRC:0.116), logistic regression (AUROC:0.903, AUPRC:0.209), and random forest (AUROC:0.910, AUPRC:0.179).

**Conclusion:**

Deep-learning-based Triage and Acuity Score predicted in-hospital mortality, critical care, and hospitalization more accurately than existing triages and acuity, and it was validated using a large, multicenter dataset.

## Introduction

Overcrowding in an emergency department (ED) has been identified as a healthcare crisis in many nations.[1,2] Triage is important in identifying vulnerable and high-risk patients among a large number of less urgent patients as ED overcrowding and delay in care are associated with increased mortality in many conditions.[3] The rapid assessment of the patient’s risk and urgency is necessary to identify high-risk patients and determine treatment priority on arrival at the ED.

The Canadian Triage and Acuity Scale (CTAS) was developed in 1999 after studying the successful National Triage Scale (NTS) from Australia.[4] The Korean Triage and Acuity System (KTAS) was developed in 2012 based on CTAS and has been used nationwide as triage since 2016 in Korea.[5] Although these Triage and Acuity Scores (TASs) help identify patients with high-risk of death, they have two limitations. First, they rely on the provider’s subjective judgement of critical care needs and pain intensity.[6,7] As a decision can be different for each provider, outcomes predicted by these TASs have high variation and low reliability.[8] Second, they can be a bottleneck in the ED patient’s flow because subjective information cannot be instantly judged and is often ambiguous. In addition, the time to judge can take more depending on the experience of the provider as subjective information is based on clinical expertise.[9] This delay is a risk to patient safety.

The Modified Early Warning Score (MEWS) is a widely used tool and overcomes two limitations using physiological parameters (systolic blood pressure, pulse rate, respiratory rate, temperature, and level of consciousness (Alert, Voice, Pain, Unresponsive).[10–14] However, it has a limitation in capturing the relationship between parameters. MEWS is the sum of the scores for each parameter, and the score for each parameter is calculated independently. For example, systolic blood pressure is not considered when calculating the score for the temperature even though the temperature is interpreted differently according to systolic blood pressure.

Machine learning (ML) based overcomes the limitation of MEWS and shows higher performance than MEWS.[15] ML is an algorithm that allows a computer to learn by itself from given data without explicitly programming (i.e., improved performance on a specific task). Until the last few years, several domains, including TAS, used ML such as logistic regression (LR) and random forest (RF).[16–18] LR finds the relationship parameters and outcome and expresses it as a linear combination of parameters. RF creates several decision trees with ensemble technique and combines the results from them. The decision tree is to build a tree-like graph (i.e., model) that predicts the outcome by learning discrete cut-points (i.e., rule). Recently, deep learning (DL) has achieved state of the art performance in several domains through deep hierarchical feature construction.[19–21] One of the most important advantages of DL compared to ML is feature learning. From a large number of data, the deep learning automatically learns the features or representations needed for given tasks such as classification and detection using several non-linear modules. In this study, we developed a Deep-learning-based TAS (DTAS) and validated that DTAS significantly outperforms existing TAS using a large, multicenter dataset.

## Methods

We conducted a retrospective observational cohort study using data from the Korean National Emergency Department Information System (NEDIS) which collected data on all visits in real time from 151 EDs in Korea. The NEDIS data was split into derivation data (January 2014-June 2016) and validation data (July-December 2016). Furthermore, we used data from the Sejong General Hospital (SGH) for external validation (January-December 2017). The hospital is a specialist cardiovascular teaching hospital, with approximately 14,000 patients visiting the ED each year. As shown in Table 1, internal and external validation data had different characteristics. We verified that DTAS was not biased towards specific characteristics through the validation of both data. The Sejong General Hospital Institutional Review Board approved this study and granted waivers of informed consent based on general impracticability and minimal harm. Patient information was anonymized and de-identified before the analysis.

**Table 1:** Baseline characteristics of the study subjects.

		NEDIS^a^ patients	SGH^a^ patients	
	Derivation data	Internal validation	External validation	*p*-value^b^
Characteristics	(n = 8,981,184)	(n = 1,986,334)	(n = 13,989)	
Study period	1/1/2014-6/30/2016	7/1/2016-12/31/2016	1/1/2017-12/31/2017	
Female, No. (%)	4,511,654 (50.2%)	1,000,513 (50.4%)	7,170 (51.3%)	0.005
Age, mean ± SD	49.9 ± 18.9	50.5 ± 19.1	51.6 ± 23.5	<0.001
Initial vital signs, mean ± SD				
Systolic BP (mmHg)	131.2 ± 23.3	131.8 ± 23.6	125.8 ± 19.4	<0.001
Diastolic BP (mmHg)	79.3 ± 13.9	79.3 ± 14.3	77.0 ± 11.3	<0.001
Heart rate (/min)	83.8 ± 16.2	84.5 ± 16.8	84.7 ± 21.2	<0.001
Respiratory rate (/min)	19.6 ± 2.7	19.5 ± 2.7	19.8 ± 3.8	<0.001
Body temperature (°C)	36.7 ± 0.7	36.8 ± 0.7	36.7 ± 0.7	0.755
Mental status, No. (%)				<0.001
Alert	8,674,058 (96.6%)	1,919,259 (96.6%)	13,770 (98.4%)	
Reacting to Voice	161,624 (1.8%)	35,781 (1.8%)	88 (0.6%)	
Reacting to Pain	113,192 (1.3%)	24,346 (1.2%)	85 (0.6%)	
Unresponsive	32,310 (0.3%)	6,948 (0.3%)	40 (0.3%)	
Arrival mode, No. (%)				<0.001
Air Transport	7,245 (0.1%)	1,675 (0.1%)	7 (0.1%)	
Ground Ambulance	2,212,231 (24.6%)	501,367 (25.2%)	3,392 (24.2%)	
Other vehicles	6,450,117 (71.8%)	1,457,125 (73.4%)	10,399 (74.3%)	
Walk in	311,591 (3.5%)	26,167 (1.3%)	185 (1.3%)	
Symptom onset to ED arrival time, No. (%)				<0.001
- 24 hours	5,394,527 (60.1%)	1,216,608 (61.2%)	8,328 (59.5%)	
24–72 hours	2,666,179 (29.7%)	583,083 (29.4%)	5,320 (38.0%)	
72 hours—7 Days	536,525 (6.0%)	111,573 (5.6%)	280 (2.0%)	
7 Days—30 Days	258,641 (2.9%)	51,045 (2.6%)	43 (0.3%)	
30 Days -	125,312 (1.4%)	24,025 (1.2%)	12 (0.1%)	
Trauma, No. (%)	2,536,815 (28.2%)	556,455 (28.0%)	2034 (14.5%)	<0.001
Korean Triage and Acuity System (KTAS), No. (%)				<0.001^d^
Level 1, Resuscitation	-^c^	16,589 (0.8%)	26 (0.2%)	
Level 2, Emergent	-^c^	140,325 (7.1%)	92 (0.7%)	
Level 3, Urgent	-^c^	721,686 (36.3%)	433 (3.1%)	
Level 4, Less urgent	-^c^	870,206 (43.8%)	4,327 (30.1%)	
Level 5, Non-urgent	-^c^	237,528 (12.0%)	9,105 (65.1%)	
Outcomes, No. (%)				<0.001
In-hospital mortality	125,219 (1.4%)	27,998 (1.4%)	150 (1.1%)	
Critical care	511,342 (5.7%)	113,775 (5.7%)	987 (7.1%)	
Hospitalization	2,433,994 (27.1%)	530,373 (26.7%)	4,337 (31.0%)	

^a^NEDIS denotes National Emergency Department Information System, SD indicates Standard Deviation, and SGH means Sejong General Hospital.

^b^The alternative hypothesis for this p-value is that there is a difference between NEDIS patients and SGH patients.

^c^Korean Triage and Acuity System has been implemented nationwide since 2016. For this reason, this column is blank.

^d^The alternative hypothesis for this p-value is that there is a difference between NEDIS internal validation patients and SGH patients.

The NEDIS data included age, sex, arrival time, chief complaint, arrival mode, initial vital signs, trauma, ED treatment result, place of hospitalization, admission result, KTAS, discharge diagnosis, etc. The study subjects were adult patients (≥18 years), and patients who were dead on arrival or had missing value were excluded.

The primary outcome was in-hospital mortality. The secondary outcome was critical care, and the tertiary outcome was hospitalization in this study. The critical care outcomes comprised of direct admission to the intensive care unit (ICU), transfer to other hospitals for ICU admission, and in-hospital mortality. The hospitalization outcomes consisted of direct admission to hospital, transfer to other hospitals for admission, and in-hospital mortality. Admitted patients who eventually die were included in the critical care outcome and the hospitalization outcome. However, each outcome was not double counted because we predicted independently for each outcome whether it would occur or not: "hospitalization or non-hospitalization," "critical care or non-critical care," and "mortality or non-mortality." We use age, sex, chief complaint, time from symptom onset to ED visit, arrival mode, trauma, initial vital signs and mental status as predictor variables (Table 1).

We developed DTAS using multilayer perceptron, a method of deep learning, with 5 hidden layers. Because there was no gain in accuracy when adding more than 5 layers, we made up 5 layers to minimize the parameters to be learned. The first to fourth layers consisted of 32, 32, 16, and 8 nodes, and applied a rectified linear activation. The last layer consisted of 1 node which represented the risk of each outcome and applied a sigmoid function. We learned DTAS as the Adam optimizer and used a binary-cross entropy as a loss function.[22] To validate our model, we used the hyperparameters of the model with the best performance on 10% of the data from the derivation data during the training process.

We compared the performance of DTAS with KTAS, MEWS, LR, and RF. KTAS has been used nationwide as triage since 2016 in Korea. MEWS is widely used as a tool to identify patients at risk of deterioration, and several studies have shown good results with MEWS in predicting poor outcomes of ED patient.[13,23,24] In the previous studies, LR and RF were the most commonly used machine learning algorithms and showed better performance than MEWS.[25–27]

We conducted a performance test exclusively for each outcome. We used the area under the receiver operating characteristic curve (AUROC) and area under the precision and recall curve (AUPRC) as the comparative measures. AUROC is one of the most used metrics in evaluating binary classifiers and shows sensitivity against 1-specificity. Compared with AUROC, AUPRC is useful with an imbalanced data like our study and show precision (i.e., 1-false positive) against recall (i.e., sensitivity).[28] With imbalanced data, in which the number of negatives outweighs the number of positives, AUROC has a limitation for evaluating the performance because the false positive rate (false positive/total real negatives) does not decrease dramatically when the total negatives are large.

## Results

A total of 11,656,559 ED visits to 151 hospitals were included in the NEDIS. We excluded 689,041 visits due to 114,368 dead on arrivals and 574,673 missing values. Study subjects comprised of 10,967,518 ED visits and the outcomes were 153,217 in-hospital death (1.4%), 625,117 critical care admissions (5.7%), and 2,964,367 hospitalization (27.0%) (Table 1). DTAS was developed using derivation data of 8,981,184 patients, and the validation study was performed using data of 1,986,334 patients on the NEDIS. External validation was performed using 13,989 visits to SGH ED, where the outcomes were 150 in-hospital death (1.1%), 987 critical care admissions (7.1%), and 4,337 hospitalizations (31.0%).

**Fig 1:**
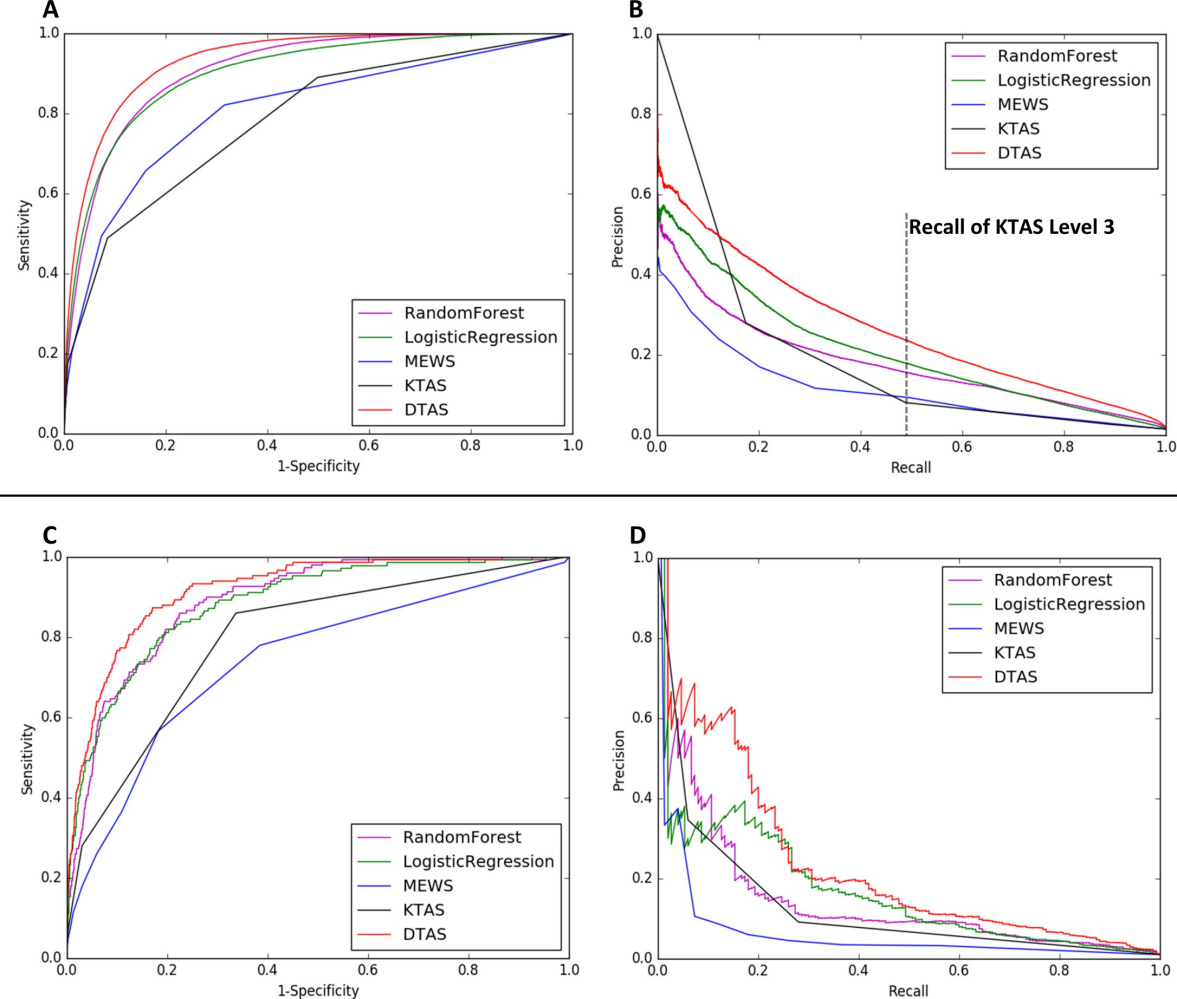
Accuracy for predicting in-hospital mortality. Fig 1 shows Receiver operating characteristic (ROC) curve and precision-recall (PR) curve for predicting in-hospital mortality. ROC curve of internal validation (A) and PR curve of internal validation (B) show that the Deep-learning-based Triage and Acuity Score (DTAS) predicted in-hospital mortality more accurately than Korean Triage and Acuity System (KTAS), Modified Early Warning Score (MEWS), Random Forest (RF), and Logistic Regression (LR) using the National Emergency Department Information System (NEDIS) data (Table 1). The ROC curve of external validation (C) and PR curve of external validation (D) demonstrated that DTAS predicted in-hospital mortality more accurately than other methods using the Sejong General Hospital (SGH) dataset. With respect to external validation, DTAS (AUROC: 0.92, AUPRC: 0.23) significantly outperformed KTAS (AUROC:0.80, AUPRC: 0.13), MEWS (AUROC: 0.74, AUPRC: 0.06), RF (AUROC: 0.89, AUPRC: 0.14), and LR (AUROC: 0.89, AUPRC:0.16).

As shown in Fig 1 and Table 2, DTAS (AUROC: 0.935, AUPRC: 0.264) significantly outperformed KTAS (AUROC: 0.785, AUPRC: 0.192), MEWS (AUROC: 0.810, AUPRC: 0.116), LR (AUROC: 0.903, AUPRC: 0.209), and RF (AUROC: 0.910, AUPRC: 0.179) with respect to in-hospital mortality. DTAS also outperformed KTAS, MEWS, LR, and RF with respect to critical care and hospitalization (Table 2). With respect to external validation, DTAS consistently showed better performance than other TASs.

**Table 2:** Accuracy for predicting in-hospital mortality, critical care, and hospitalization.

	AUROC	(95% CI)	*p*-value^a^	AUPRC	(95% CI)	*p*-value^a^
**Predicting In-hospital mortality**						
DTAS	0.935	(0.935–0.936)	-	0.264	(0.263–0.265)	-
KTAS	0.785	(0.785–0.786)	<0.001	0.192	(0.192–0.193)	<0.001
MEWS	0.810	(0.809–0.810)	<0.001	0.116	(0.116–0.117)	<0.001
RF	0.910	(0.910–0.910)	<0.001	0.179	(0.178–0.180)	<0.001
LR	0.903	(0.902–0.903)	<0.001	0.209	(0.208–0.210)	<0.001
**Predicting Critical care**						
DTAS	0.894	(0.894–0.895)	-	0.460	(0.460–0.460)	-
KTAS	0.797	(0.797–0.797)	<0.001	0.376	(0.375–0.376)	<0.001
MEWS	0.726	(0.725–0.726)	<0.001	0.236	(0.235–0.236)	<0.001
RF	0.822	(0.821–0.822)	<0.001	0.338	(0.337–0.338)	<0.001
LR	0.818	(0.818–0.818)	<0.001	0.349	(0.349–0.350)	<0.001
**Predicting hospitalization**						
DTAS	0.804	(0.803–0.804)	-	0.654	(0.654–0.655)	-
KTAS	0.681	(0.681–0.681)	<0.001	0.525	(0.524–0.525)	<0.001
MEWS	0.614	(0.614–0.614)	<0.001	0.444	(0.444–0.444)	<0.001
RF	0.738	(0.738–0.738)	<0.001	0.557	(0.557–0.558)	<0.001
LR	0.713	(0.713–0.713)	<0.001	0.531	(0.531–0.531)	<0.001

CI denotes confidence intervals, DTAS Deep-learning-based Triage and Acuity Score, KTAS Korean Triage and Acuity System, and MEWS Modified Early Warning Score, RF Random Forest, and LR Logistic Regression.

^a^The alternative hypothesis for this p-value is that there is a difference the between area under the curve of DEWS and other methods.

As shown in Fig 1, the sensitivity of KTAS level 3 was 0.49 for predicting in-hospital mortality. At this point, the precisions of DTAS, KTAS, MEWS, RF, and LR were 0.24, 0.08, 0.09, 0.16, and 0.18, respectively.

## Discussion

We found that DTAS showed the best performance for predicting in-hospital mortality, critical care, and hospitalization based on a large, multicenter dataset. DTAS can reduce a false positive by 67% compared to KTAS. This reduction in false positives increases the practical applicability of DTAS.

Several previous studies attempted to predict outcomes of ED patients. Taylor et al. reported a new random forest method for predicting in-hospital mortality of emergency department patients with sepsis.[29] Ong et al. reported a conventional machine learning model for predicting cardiac arrest in critically ill patients presenting to the ED.[30] But two studies used small population and did not perform multicenter validation. The performance of algorithms based on given data rather than medical knowledge, such as machine and deep learning, is not guaranteed in other environments. The algorithms can memorize only the characteristics of derivation data. Because they learn the relationship between the predictor variables and outcome from only given data. Wolpert explains the No Free Lunch theorem; if optimized in one situation, a model cannot produce good results in other situations.[31]

We used the national big data NEDIS to develop and validate DTAS, and the subjects of this study were those who visited ED across the whole country. Therefore, DTAS learned the characteristics of all patients nationwide rather than any particular area. However, DTAS can be biased to the average of NEDIS (i.e., overfitting). So, we verified DTAS using SGH (external validation) which had different characteristics from NEDIS. Through multicenter validation, we showed that the performance of DTAS was not biased towards specific characteristics and guaranteed in other environments.

Most patients do not experience rare events such as in-hospital mortality and critical care (i.e., imbalanced data). In this environment, AUPRC is a more important metric than AUROC. With imbalanced data, in which the number of negatives outweighs the number of positives, AUROC has a limitation for evaluating the performance because the false positive rate (false positive/total real negatives) does not decrease dramatically when the total negatives are large. AUPRC, on the other hand, is suitable for imbalanced data, as they consider the fraction of true positives among positive predictions.[32] Although DTAS can reduce false positives by 67% compared to KTAS, the AUROC of DTAS is only 19% higher than the AUROC of KTAS for predicted in-hospital mortality. On the other hand, AUPRC of DTAS is 38% higher than AUPRC of KTAS.

Unfortunately, traditional triage tools are complex scoring methods that require detailed history taking and physical exams (e.g., pain score, evidence of dehydration, pitting edema, and blood sugar test result), and judgment based on clinical experience (e.g., expected emergency department resource).[4,7] These tools require considerable time for triage and are of limited use in resource-constrained settings of circumstances in which junior triage provider, who have limited training and experience, practice.[9,33] Numerous studies concluded that dedicating a senior doctor in triage reduced the waiting time for patients to see a doctor, decreased the LOS, and lowered the proportion of leftover patients without being seen.[33,34] However, this solution requires enormous cost.[35]

On the other hand, DTAS requires only age, sex, chief complaint, symptom to visit time, arrival mode, trauma or not, initial vital sign, and mental status as input parameters. This allows DTAS to have three strengths. First, outcomes predicted by DTAS have low variation and high reliability because input parameters are basic information with low inter-physician variation. Second, input parameters do not require expert judgment and can be collected very quickly, it would be of great value in a resource-constrained ED setting. Third, parameters of DTAS can be checked in a pre-hospital setting and DTAS score can be calculated in pre-hospital transport and out-of-hospital situations. Therefore, DTAS has the potential to make the process of pre-hospital emergency medical service (EMS) and ED efficient. Our next area of focus for research is the prospective study of EMS and ED triage to verify the performance and efficiency of DEWS.

## Conclusion

Deep-learning-based Triage and Acuity Score predicted in-hospital mortality, critical care, and hospitalization more accurately than existing triages and acuity, and it was validated using a large, multicenter dataset.
